# Prediction of Healing Performance of Autogenous Healing Concrete Using Machine Learning

**DOI:** 10.3390/ma14154068

**Published:** 2021-07-21

**Authors:** Xu Huang, Mirna Wasouf, Jessada Sresakoolchai, Sakdirat Kaewunruen

**Affiliations:** 1 Laboratory for Track Engineering and Operations for Future Uncertainties (TOFU Lab), School of Engineering, University of Birmingham, Birmingham B152TT, UK; XXH689@student.bham.ac.uk (X.H.); JSS814@student.bham.ac.uk (J.S.); 2 Department of Civil Engineering, School of Engineering, University of Birmingham, Birmingham B152TT, UK; MXW913@alumni.bham.ac.uk

**Keywords:** machine learning, autogenous healing concrete, self-healing concrete, enhanced autogenous healing concrete, hyperparameters tuning, genetic algorithm

## Abstract

Cracks typically develop in concrete due to shrinkage, loading actions, and weather conditions; and may occur anytime in its life span. Autogenous healing concrete is a type of self-healing concrete that can automatically heal cracks based on physical or chemical reactions in concrete matrix. It is imperative to investigate the healing performance that autogenous healing concrete possesses, to assess the extent of the cracking and to predict the extent of healing. In the research of self-healing concrete, testing the healing performance of concrete in a laboratory is costly, and a mass of instances may be needed to explore reliable concrete design. This study is thus the world’s first to establish six types of machine learning algorithms, which are capable of predicting the healing performance (HP) of self-healing concrete. These algorithms involve an artificial neural network (ANN), a k-nearest neighbours (kNN), a gradient boosting regression (GBR), a decision tree regression (DTR), a support vector regression (SVR) and a random forest (RF). Parameters of these algorithms are tuned utilising grid search algorithm (GSA) and genetic algorithm (GA). The prediction performance indicated by coefficient of determination (R^2^) and root mean square error (RMSE) measures of these algorithms are evaluated on the basis of 1417 data sets from the open literature. The results show that GSA-GBR performs higher prediction performance (R^2^_GSA-GBR_ = 0.958) and stronger robustness (RMSE_GSA-GBR_ = 0.202) than the other five types of algorithms employed to predict the healing performance of autogenous healing concrete. Therefore, reliable prediction accuracy of the healing performance and efficient assistance on the design of autogenous healing concrete can be achieved.

## 1. Introduction

Concrete is a commonly used material for construction and aesthetic purposes. It is estimated that about three tons of concrete are used per person per year, with that amount doubled when other materials are added for construction [1]. Concrete can be classified into various types, obtained by varying the mix proportions and cement type to achieve different qualities for the intended purposes. Because of its dire importance, its properties are always under investigation and testing to make it more efficient and last longer. Concrete cracking is one of the main problems with the material for the possible seeping of harmful substances and internal damage to the structural members’ components. The formation of fractures in this material is a foreseeable outcome, but precautions may be carried out to minimise its adverse effects on the structures [2]. The most commonly occurring and threatening form of reinforced concrete deterioration is pitting corrosion [3]. The presence of three substances is responsible for the electromechanical process resulting in corrosion—a conductive medium, such as steel from the reinforcement, a source of moisture and, finally, oxygen. The process may be as indicated by the reaction in Equation (1) [4].

(1) $$4\mathrm{Fe}+3O_{2}+2{\mathrm{nH}}_{2}O=2{\mathrm{Fe}}_{2}O_{3}\times {\mathrm{nH}}_{2}O\left(↓\right)$$

The extent of corrosion was investigated that manifests different cracking widths in the reinforced concrete. Corrosion was observed on the rebars of test specimens with varying crack thicknesses. It was found that corrosion did occur in the reinforcement of samples with 400 µM crack girths. No corrosion was detected in concrete attaining a maximum crack width of 100 µM, even after 365 days have elapsed from the commencement of the trials [5]. Cathodic protection may be employed to protect the inner materials from this destructive reaction. The method and effects of inserting rods within the concrete structure are studied, which are composed of more reactive materials, such as Zinc or Magnesium acting as sacrifices by attracting the reactive ions and diverting the corrosive response away from the reinforcement. Moreover, it was proven that the absence of any of the three components is satisfactory for preventing the rust from eating away at the reinforcement [6]. Knowing this promoted the exploration of means to minimise the exposure to air—the main cause of cracks. A guaranteed, albeit expensive, provision is coating the reinforcement with protective films. Costs may be reduced by investigating which surfaces are most likely to be exposed to moisture or air. The rods anticipated to be placed in close vicinity to said surfaces would be engulfed with the water-repelling material prior to casting the concrete [7]. Painting the of structural members after being cast is another route followed when maintaining their integrity. The coating acts as a shield preventing moisture and water from seeping into the load bearing materials. However, this requires constant maintenance to ensure that intended performance is preserved. Numerous other measures have been instituted to reduce the adverse effects of fissures and corrosion on reinforced concrete [8]. It can be concluded that limiting the oxygen supply to the other components halts the corrosion. Additionally, it is considered relatively less costly to install precautionary measures and control the presence of cracks before casting as compared with maintaining and fixing the damaged members after time has elapsed [3].

Concrete has been found to have self-healing capacities that seal microcracks depending on various testing conditions and concrete mix proportions [9]. Self-healing concrete is classified into two categories, autogenous healing concrete and agent-based healing concrete. The naturally occurring ability which heals cracks on the basis of ingredients in concrete matrix is referred to as autogenous healing. Therefore, autogenous healing concrete consists of intrinsic healing concrete and enhanced autogenous healing concrete. On the other hand, agent-based healing concrete indicates the healing concrete based on employing healing agents, such as polymer or bacteria.

### 1.1. Intrinsic Healing Concrete

**Figure 1:**
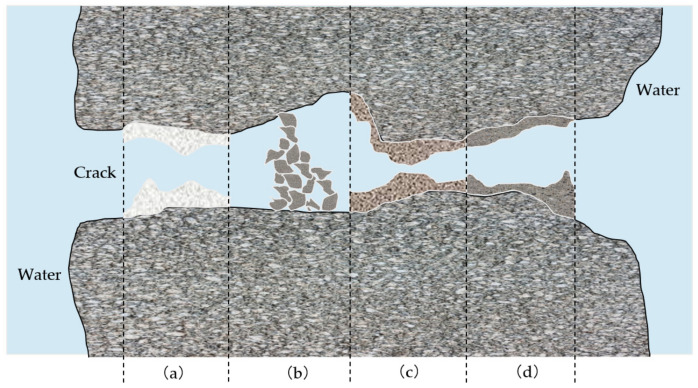
Illustration of the four reactions aiding in self-healing of concrete: (**a**) white precipitation formation; (**b**) loose particles blockage; (**c**) rehydration of unreacted cement particles; (**d**) swelling particles blockage by ASR.

The mechanisms of autogenous healing concrete can be concluded as follows, shown in Figure 1. Firstly, calcium carbonate crystals form after the CO_2_ reacts with calcium ions (white precipitation), exhibited in Figure 1a. Secondly, impurities or debris from water may act as a wall to prevent harmful substances from penetrating the concrete and affecting its structural integrity, shown in Figure 1b. Thirdly, cement that has not hydrated during the mixing and setting of the concrete may hydrate after it has hardened, shown in Figure 1c. Fly ash and slag may also ‘bloom late’, i.e., hydrate at a late stage of the concrete curing period. Finally, aggregate silicate reaction (ASR) causes the swelling of the concrete, indicated in Figure 1d. The growth may progress into the cracks and seal them. The member’s ingress may be averted by forming a barrier between the offensive materials and the concrete within the structural member. The processes and the reasons of the substance formed by the mechanisms aforementioned are explained as three aspects below [10,11].

#### 1.1.1. Precipitate Formation

A combination of $\mathrm{Ca}{\left({\mathrm{HCO}}_{3}\right)}_{2}$ crystals that come from water and $\mathrm{Ca}{\left(\mathrm{OH}\right)}_{2}$ particles from concrete (calcium hydrogen carbonate and calcium hydroxide, respectively) react with one another upon contact [12], triggering this interaction that forms calcium carbonate compound (${\mathrm{CaCO}}_{3}$), which is the white precipitate that is illustrated in Figure 1a. The chemical reactions are presented in the chemical reactions denoted as Equations (2)–(5) [13,14]. The formed crystalline substance protects the structural members from harmful elements by coating the microcracks. This prevents elements that may have otherwise been able to trickle through the cracks and cause ingress to the structural members.

(2) $${\mathrm{CO}}_{2}+H_{2}O↔H_{2}{\mathrm{CO}}_{3}$$

(3) $$H_{2}{\mathrm{CO}}_{3}+\mathrm{Ca}{\left(\mathrm{OH}\right)}_{2}={\mathrm{CaCO}}_{3}↓+H_{2}O$$

(4) $$H_{2}{\mathrm{CO}}_{3}+{\mathrm{CaCO}}_{3}↔\mathrm{Ca}{\left({\mathrm{HCO}}_{3}\right)}_{2}$$

(5) $$\mathrm{Ca}{\left({\mathrm{HCO}}_{3}\right)}_{2}+\mathrm{Ca}{\left(\mathrm{OH}\right)}_{2}=2{\mathrm{CaCO}}_{3}↓+2H_{2}O$$

#### 1.1.2. Continued Hydration

The hydration of previously un-hydrated cement particles present in the mixture aids in sealing the small cracks. Continuous hydration autogenous healing utilises the reaction between the cement and the water, producing a gel that swells and blocks the narrow pathway paved by fissures, thus reducing the flow rate within the sample [15,16]. The related reactions are exhibited in Equations (6)–(9). It has been proven that younger concrete possesses more self-healing capacity than the old due to the continued hydration occurrence.

(6) $$C_{3}S+6H_{2}O\to C_{3}S_{2}\cdot 3H_{2}O+3\mathrm{Ca}{\left(\mathrm{OH}\right)}_{2}$$

(7) $$C_{2}S+4H_{2}O\to C_{3}S_{2}\cdot 3H_{2}O+3\mathrm{Ca}{\left(\mathrm{OH}\right)}_{2}$$

(8) $$C_{3}A+6H_{2}O\to C_{3}A\cdot 6H_{2}O$$

(9) $$4C_{4}\mathrm{AF}+2\mathrm{Ca}{\left(\mathrm{OH}\right)}_{2}+10H_{2}O\to C_{3}A\cdot 6H_{2}O+C_{3}F\cdot 6H_{2}O$$

#### 1.1.3. ASR

ASR generally has destructive repercussions. The internal effect of the interaction between alkali, found in sand or gravel used in the mixture, and cement produces an expansive gel that swells within the member [17,18]. Building on the expansion ability of aggregates, if the ASR reaction is monitored and limited, future cracking can be prevented by transforming the distressing reaction into one that results in another type of sealant by ASR [19].

### 1.2. Enhanced Autogenous Healing Concrete

#### 1.2.1. Mineral Additions

Incorporating mineral additions with cementitious materials is an effective method of improving HP of self-healing concrete, and the most commonly employed minerals are fly ash, blast furnace slag and silica fume [16,20]. The healing ability of enhanced autogenous healing concrete is attributed to the pozzolanic reaction between mineral additions and cementitious materials because the pozzolanic reaction can stimulate the hydration of cementitious materials to form C-S-H for healing cracks.

#### 1.2.2. Crystalline Admixtures

Crystalline admixtures are commercial types of healing materials whose ingredients are confidential. According to open literature, sulphur trioxide and sodium oxide are revealed as constituents of crystalline admixtures [21,22]. The healing mechanism of crystalline admixtures can be concluded as such Ca ions from crystalline admixtures react with ${\mathrm{CO}}_{3}^{2-}$ and ${\mathrm{HCO}}_{3}^{-}$ existing in cracks, producing calcium carbonate for healing cracks, as given by Equations (2)–(5).

#### 1.2.3. SAP

Superabsorbent polymers (SAP) are white powder or scale-like ranging from mi-cros to millimetres. The healing steps of concrete with SAP can be concluded as follows. Firstly, the water inside SAP is released into concrete matrix to promote further hydration of cementitious materials. As a result, C-S-H, which is able to heal cracks, can be formed during the further hydration of cementitious materials. Secondly, SAP particles can expand to seal cracks when water penetrates concrete through cracks [23,24,25,26].

#### 1.2.4. Fibre

Concrete containing fibre has been paid more attention to because of its excellent ability to crack resistance. The healing mechanisms of concrete with fibre can be drawn as follows. Firstly, fibre can effectively limit the crack width of concrete matrix under varying conditions and offer the bridging force to enhance the healing ability of concrete with fibre. Secondly, fibre can be the cores of precipitations to stimulate the formation of healing products. The results of published articles related to fibre healing concrete demonstrated that various types of fibre, different geometry including diameter, length and tensile strength of fibre exhibited a significant influence on the healing performance of concrete [27,28].

### 1.3. Agent-Based Healing Concrete

Healing methods involving external interference, namely agent-based healing concrete, were investigated. According to the research on agent-based healing concrete, calcium carbonate for healing cracks was induced by applying bacteria, such as ureolytic bacteria, aerobic bacteria and nitrifying bacteria, and their nutrients onto the test specimen. The general mechanism of agent-based healing concrete can be attributed to the following steps. Firstly, capsules containing healing agents are incorporated into the mixture and spread throughout the cast concrete samples. Secondly, when cracks are formed, the damage would cause the aforementioned capsules to release the entrained substances within them, thus healing the cracks [29,30,31].

However, better healing performance of agent-based healing concrete is mentioned. Up to 970 µM wide of cracks were reported to be healed by employing agent-based healing concrete [30]. Two main drawbacks of agent-based healing concrete are noted. Firstly, using capsules as carriers with non-biodegradable materials such as plastics and other synthetics to distribute healing agents, such as polymers or bacteria, is less environmentally friendly [32]. Secondly, utilising agent-based healing method to heal cracks is dramatically more costly than autogenous healing concrete. Considering the two inevitable disadvantages, autogenous healing concrete is studied in this paper.

Machine learning (ML) has been broadly utilised to solve regression, clustering and classification problems according to the information dug out from massive data sets. The reason why ML has the ability to figure out these problems can be attributed to ML being able to obtain new knowledge by means of learning from existing information imitating human learning behaviour. To date, two studies attempted to predict HP of self-healing concrete. One study carried out by Ramadan and Nehdi was involved in predicting HP of intrinsic healing concrete employing ANN whose parameters were optimised by GA [33]. In a study of conducted by Zhuang and Zhou, it was shown that HP of healing concrete containing non-ureolytic bacteria can be accurately predicted by the GBR ML model [34]. In both studies, the initial cracking time, the initial cracking width and healing materials information are recognised as inputs. However, due to complicated healing mechanisms of self-healing concrete, it is necessary to consider all influencing factors of HP of self-healing concrete.

In this paper, 16 influencing factors are utilised for the first time to predict HP of autogenous healing concrete. Firstly, 1417 experimental data sets in total of autogenous healing concrete are collected from eight open literature. Then, 16 variables are set as the inputs, and the healing performance of autogenous healing concrete is recognised as the sole output. Subsequently, unprecedented six ML algorithms are employed to build various ML models, and two types of hyperparameters optimisation methods are applied to tune the parameters of each ML algorithm. After that, the prediction performance and the prediction accuracy of each ML model are demonstrated and then compared using R^2^ and RMSE. Finally, sensitive analysis on the optimal ML model is conducted.

## 2. Materials and Methods

### 2.1. Data Collection

In this study, 1417 experimental data sets related to HP of autogenous healing concrete are collected from eight open literature published from 2000 to 2020 shown in Table 1. A total of 1417 data sets in this research are randomly split into two data sets with a ratio at 8:2. 80% data sets are randomly selected and employed to train machine learning models, and the rest of the data sets are utilised as the testing data sets to inspect the generalisation capacity of the machine leaning methods. In order to input collected data sets in ML models, the representation numbers of healing materials, cement types and healing conditions are listed in Table 2. In order to accurately predict HP of autogenous healing concrete, all influencing factors regarding HP of autogenous healing highlighted by several academic literature are calculated as inputs in this study [35]. Concerning the inputs, five of them explain the influencing factors of healing materials while others describe the influencing factors related to cementitious materials displayed in Table 3. Therefore, the influencing factors of healing materials include types of healing materials (HM), dosages of healing materials (DOHM), fibre diameters (FD), fibre length (FL), fibre tensile strength (FTS), the initial cracking data (CD), the time for healing (TH), the healing condition (HC) and the initial cracking width (CW), and the influencing factors of cementitious materials are the amount of cement (CM), cement types (CT), the amount of superplasticizer (S), the amount of fine aggregates (FA), the water-binder ratio (WB), the amount of fly ash (FAS) and the amount of slag (SG). Besides, HP of various types of autogenous healing concrete calculated by changes in the crack width and the resonance frequency is treated as the output.

### 2.2. Data Normalisation

It is essential to normalise data for improving the working efficiency and the prediction performance of machine learning models. Therefore, the data are normalised between [0,1] before inputting data into machine learning models by employing Equation (10) [40].

(10) $$Yn=\frac{y-y_{min}}{y_{max}-y_{min}}$$

where *Yn* is the normalised data, *y* represents the data before normalisation, and *y_min_* and *y_max_* are the minimum and maximum data before normalisation.

### 2.3. Types of ML Algorithms

In this section, six ML algorithms are introduced from articles related to concrete properties prediction to predict HP of autogenous healing concrete. Therefore, ANN, kNN, DTR, SVM are classified as the single ML algorithms. GBR and RF are recognised as ensemble ML algorithms by employing bagging or boosting strategy to improve the prediction performance and overcome the overfitting problem. Detailed information of these algorithms can be accessed in other open literature [41,42,43,44,45,46,47,48]. These types of algorithms were utilised for predicting concrete properties. For instance, ANN models demonstrated talented ability (R^2^ = 0.9185) for predicting the compressive strength of concrete with recycled aggregate. Furthermore, DTR and GBR models were employed to predict the mechanical properties of hydraulic concrete. The results showed that GBR models demonstrated better prediction performance than DTR models. The R^2^ of GBR models for predicting the compressive strength, ultimate tensile strain, elastic modulus, dry shrinkage rate and splitting tensile strength were 0.951, 0.858, 0.934, 0.922 and 0.929 respectively.

### 2.4. Hyperparameters Tuning

#### 2.4.1. GA

GA is a probabilistic searching algorithm and an intelligence solution inspired by biological evolution processes. Each individual of a population in GA is called a chromosome. A certain proportion of chromosome among a population is selected as the next generation to continuously iterate until the global optimal chromosome is found in accordance with the fitness degree of each chromosome [49,50].

#### 2.4.2. GSA

GSA is an optimisation method to analyse all possible cases in the constraint range. The processes to conduct GSA can be concluded as follows [51,52]:

- The searching scope and length are confirmed, and then, the searching grid is generated.
- The node in the searching grid with the highest accuracy and the lowest coefficient penalty calculated by K-fold validation is defined as the node which can output the best parameter value.

Characteristics of GA and GSA are summarised in Table 4 [53,54].

### 2.5. Prediction Performance Evaluation

RMSE shown in Equation (11) is elected as the governing factor in determining the accuracy of the predictive models as it calculates the square root of the error between the predicted and observed values for all the values. The lower the RMSE, the better the fit and the more accurate the predictions [55]. R^2^ calculated by Equation (12) has also been noted to evaluate the prediction performance of ML models. Its output ranges from zero to one, one indicating a perfect model [56].

(11) $$\mathrm{RMSE}=\sqrt{\frac{\sum_{i=1}^{n}{\left(y_{i}^{\prime }-y_{i}\right)}^{2}}{n}}$$

(12) $$R^{2}=1-\frac{\sum_{i=1}^{n}{\left(y_{i}^{\prime }-y_{i}\right)}^{2}}{\sum_{i=1}^{n}{\left(y_{i}^{\prime }-\bar{y}\right)}^{2}}$$

where n indicates the number of samples, while the differences in predicted and experimental values are demonstrated as $y_{i}^{\prime }-y_{i}.$

## 3. Results and Discussion

### 3.1. R^2^ and RMSE of ML Models

**Figure 2:**
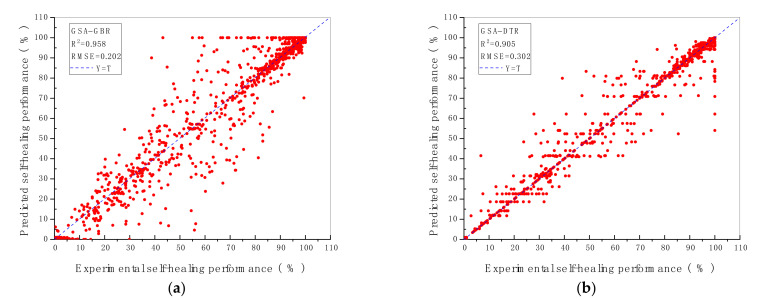

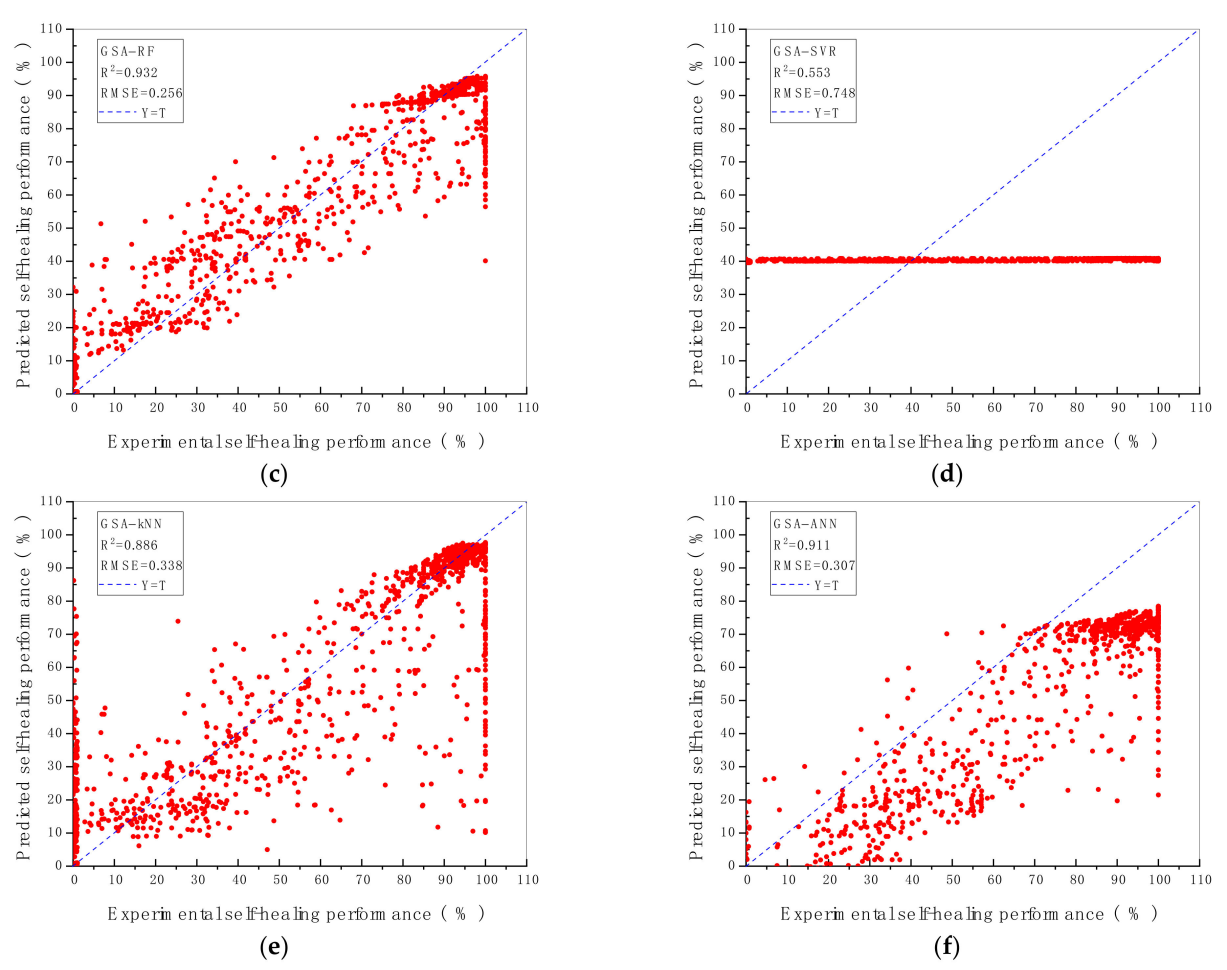
R^2^ and RMSE value of ML models with (**a**) GBR; (**b**) DTR; (**c**) RF; (**d**) SVR; € kNN; (**f**) ANN, tuned by GSA.

**Figure 3:**
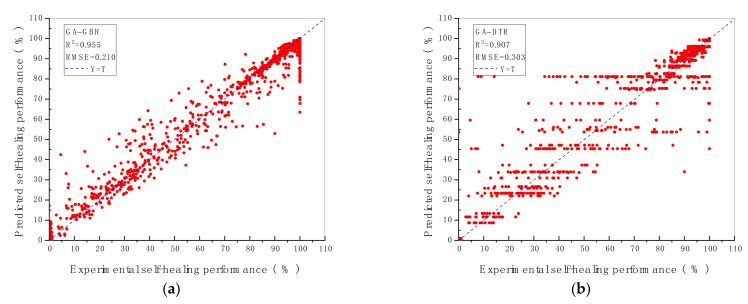

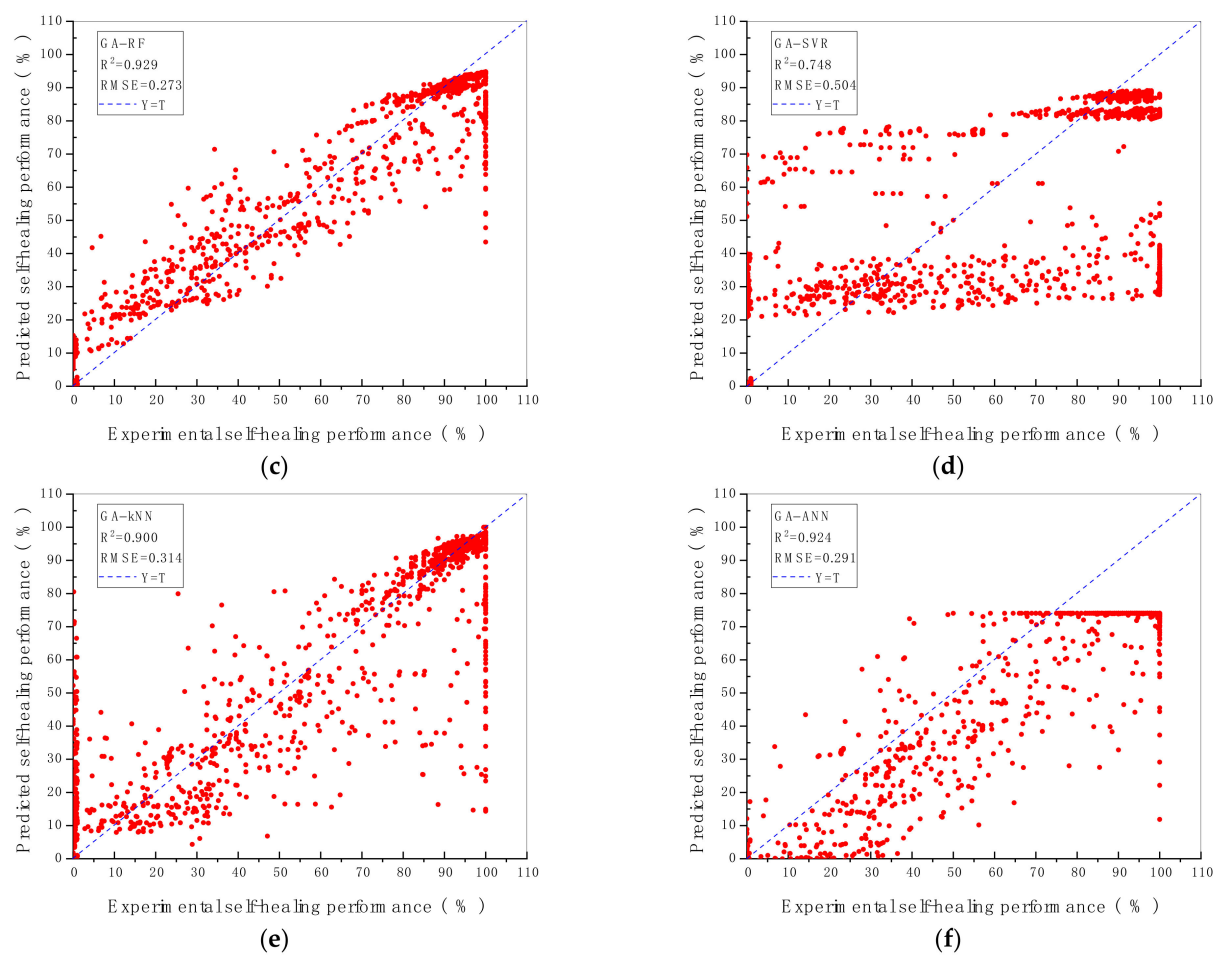
R^2^ and RMSE value of ML models with (**a**) GBR; (**b**) DTR; (**c**) RF; (**d**) SVR; (**e**) kNN; (**f**) ANN, tuned by GA.

**Figure 4:**
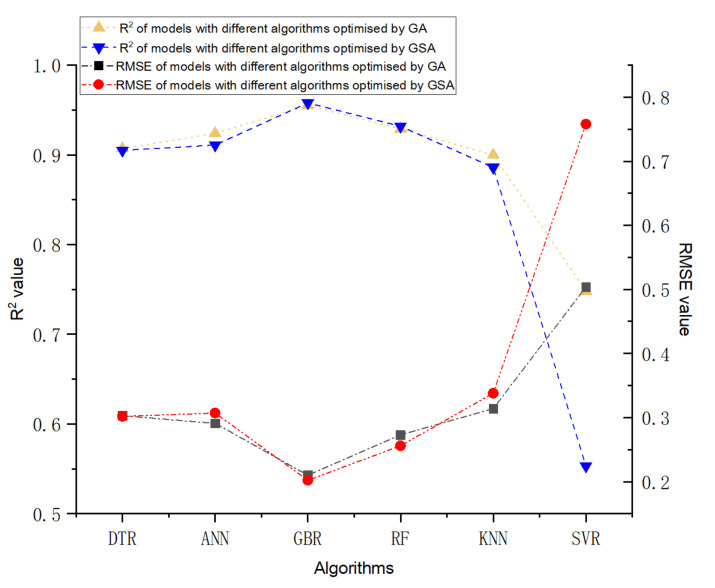
The differences of R^2^ and RMSE value of six types of ML models tuned by GSA and GA.

The prediction performance and the prediction accuracy of six machine algorithms with two hyperparameters optimisation methods are indicated by R^2^ and RMSE value shown in Figure 2 and Figure 3. As demonstrated in Figure 2 and Figure 3, the vertical axis represents the predicted self-healing performance in % output from the machine learning models, while the horizontal axis represents the experimental self-healing performance in % of 1417 test data instances collected from the open literature. Each red point shown in Figure 2 and Figure 3 stands for the predicted and the experimental self-healing performance of each specific test instance. Moreover, parameters of ML models tuned by GA and GSA are exhibited in Table 5. The model with the highest R^2^ value and the lowest RMSE value is recognised as the best ML model for predicting HP of autogenous healing concrete. What can be clearly seen in Figure 4 is the GBR model whose parameters are tuned by employing GSA (GSA-GBR), showing higher R^2^ (0.958) and lower RMSE (0.202) than other types of ML algorithms. As is shown in Figure 4, the R^2^ and RMSE values of the GA-GBR model are 0.955 and 0.210, respectively which are similar to those of GSA-GBR. The R^2^ and RMSE of GSA-RF and GA-RF are (0.932, 0.256) and (0.929, 0.273) accordingly, which indicate a slight low prediction performance and prediction accuracy than those of GSA-GBR and GA-GBR. In addition, GSA-DTR, GA-DTR, GSA-ANN, GA-ANN, GSA-kNN and GA-kNN models demonstrate fluctuation of R^2^ and RMSE values around (0.900, 0.300) which are (0.905, 0.302), (0.907, 0.303), (0.911, 0.307), (0.924, 0.291), (0.886, 0.338) and (0.900, 0.314), respectively. What is striking in Figure 4 is the dramatical drop in R^2^ and RMSE of GSA-SVR and GA-SVR. The R^2^ and RMSE values of GSA-SVR and GA-SVR are (0.553, 0.758) and (0.748, 0.504) accordingly. GA performs the better optimisation ability than GSA to enhance the prediction performance of SVR in this paper. To sum up, GSA-GBR is defined as the optimal algorithm to explain the relationship between 16 variables and HP of autogenous healing concrete.

**Figure 5:**
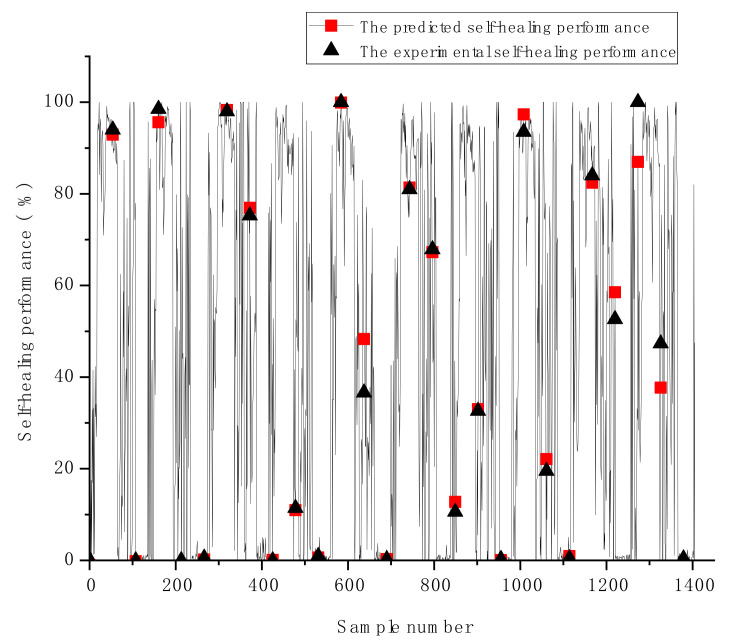
The differences of the predicted and experimental self-healing performance of the GSA-GBR model.

Additional display of the predicted HP against the individual experimental samples for the GSA-GBR model is exhibited in Figure 5. The presented plots in Figure 5 demonstrate the comparison of the experimental HP and the predicted HP of each sample. The slight differences between the predicted and experimental HP of the GSA-GBR model are shown in Figure 5. The GSA-GBR model performs reasonable prediction results for most samples, excluding few samples with relatively significant deviations (i.e., the worst predicted sample shown in Figure 5 is the sample 1295 with 0.12 of the deviation). Overall, the GSA-GBR model is emphasised as a good fit for the HP prediction of autogenous healing concrete. The reasons to explain why GSA-GBR has better prediction performance and accuracy than other types of ML algorithms with various types of optimisation methods can be summarised in two aspects. Firstly, GBR is an ensemble algorithm based on boosting strategy and DTR. Therefore, DTR has an excellent prediction performance for predicting HP of autogenous healing concrete. The overfitting problem of GBR is evitable because multiple decision trees are combined by employing the gradient boosting method to reduce the variance of decision trees. Secondly, although GSA is a time-consuming optimisation algorithm, it can find the optimal global solution.

### 3.2. Sensitive Analysis

**Figure 6:**
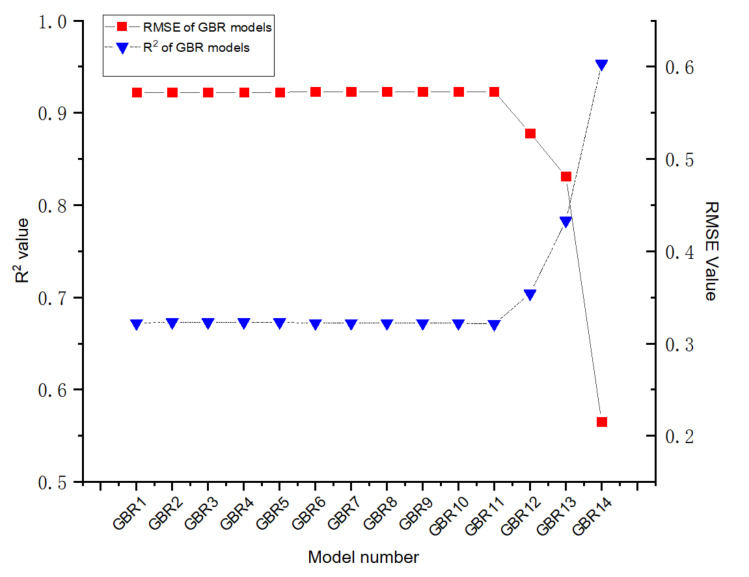
R^2^ and RMSE of GSA-GBR models (1–14).

Sensitive analysis is a type of uncertainty analysis employed to explain the results of ML according to the analysis of the impact of changed inputs on the outputs. It is significant for sensitive analysis to explore the relationship between the changes in the number of inputs and the output [57]. In this paper, the impact of 16 inputs on HP is analysed utilising ML models with GSA-GBR. Fourteen combinations of ML models with GBR are evaluated and displayed in Table 6 and Figure 6. Therefore, GBR1 represents the basic components of cementitious materials, such as the amount of cement and cement types. In GBR models 2–13, each new input is progressively counted into for analysing the influence. Therefore, GBR14 contains 14 inputs.

Figure 6 demonstrates the prediction performance and the prediction accuracy of ML models with GBR containing changed numbers of inputs. Importantly, the results shown by GBR 14 demonstrate the highest prediction performance, because GBR14 consists of all inputs. GBR1 performs the lowest prediction performance whose measures are 0.672. It is noticeable that no significant differences of the prediction performance are found between GBR models from 1 to 11. The prediction performance and the prediction accuracy of GBR models keep constant at 0.672 and 0.573, respectively, following the increasing number of inputs, as displayed by R^2^ and RMSE of GBR models 1–11. The R^2^ value of GBR models 11 and 12 increases from 0.672 to 0.704, while a decline of the RMSE from 0.573 to 0.528 is exhibited between GBR model 11 and GBR model 12. Subsequently, the R^2^ value of GBR13 rises moderately from 0.704 to 0.783. At the same time, a lesser decline of RMSE from 0.528 to 0.481 of GBR13 is demonstrated. Finally, what stands out is the rapid growth of R^2^ value of GBR14, which soars from 0.783 to 0.958. Meanwhile, the RMSE of GBR14 plummets from 0.481 to 0.202. In summary, CW, CD and TH have higher influence on HP of autogenous healing concrete, which means that they are the most significant inputs that should be paid more attention to in ML models to achieve higher prediction performance. Therefore, TH is the input which has the highest impact on HP of autogenous healing concrete.

## 4. Conclusions

This study is the world’s first to predict HP of autogenous healing concrete employing ML models using six kinds of advanced algorithms. ML models are employed to explain the relationship between 16 inputs and HP. Meanwhile, GSA and GA hyperparameter tuning methods are utilised to optimise the parameters of the ML models.

With regards to the R^2^ and RMSE values of ML models, obvious findings to emerge from this paper can be concluded as follows.

- This paper identifies that the GSA-GBR ML model has the best performance to predict HP of autogenous healing concrete, as indicated by the R^2^ value and the RMSE value (0.958 and 0.202, respectively) of GSA-GBR model. On the basis of the R^2^ value and the RMSE value, it can be attributed that the GSA-GBR ML model has an excellent ability for predicting HP of autogenous healing concrete using the 16 inputs.
- The R^2^ and the RMSE values of other ML models with five types of algorithms (SVR, RF, ANN, kNN and DTR) optimised by two kinds of hyperparameter tuning methods (GA and GSA) are compared with that of GSA-GBR. The results reveal that GSA has a better optimisation ability than GA on ML models based on DTR.
- The results of the sensitive analysis indicate that CW, CD and TH demonstrate stronger correlation of HP prediction of autogenous healing concrete than other inputs. Most importantly, CW, CD and TH have higher impact on HP prediction of autogenous healing concrete than healing materials characteristics.
- With respect to the future work, the healing performance of agent-based healing concrete can be investigated employing the latest and promising machine learning algorithms.

## Figures and Tables

**Table 1:** Experimental data source.

Data Source	Numbers of Data
Gagne and Argouges, [36]	60
Homma et al., [37]	22
Homma et al., [27]	12
Sisomphone et al., [21]	462
Tittelboom et al., [20]	343
Ozbay et al., [38]	67
Yang et al., [39]	51
Kan and Shi, [28]	400

**Table 2:** The representation of numbers of healing materials, the cement type and healing conditions.

Number	Representation
0	None
1	Calcium sulfoaluminate based expansive additive-α (CSA-α)
2	Crystalline additive
3	Calcium sulfoaluminate based expansive additive-β (CSA-β)
4	PVA fibre
5	Polyethene fibre
6	Steel cord
7	Portland Cement of Grade 42.5
8	Portland Cement of Grade 52.5
9	Ambient water condition
10	Ambient air condition
11	Wet-dry cycles

**Table 3:** The range of the 16 inputs and the output.

Variables	Unit	Minimum	Maximum
CM	(%)	0.1070	0.7140
CT	-	1.0000	2.0000
S	(%)	0.0000	0.0450
FA	(%)	0.0000	0.4420
WB	-	0.2500	0.6030
FAS	(%)	0.0000	0.6590
SG	(%)	0.0000	0.6071
HM	-	0.0000	6.0000
DOHM	(%)	0.0000	0.0310
FD	um	0.0000	400.0000
FL	um	0.0000	32000.0000
FTS	MPa	0.0000	2850.0000
CD	days	3.0000	180.0000
TH	days	0.0000	150.0000
HC	-	1.0000	3.0000
CW	um	0.0000	402.0000
HP	(%)	0.0000	100.0000

**Table 4:** Characteristics of GA and GSA.

Optimisation Algorithms	Drawbacks	Advantages
GA	GA requires sophisticated coding.	GA has good robustness in searching for the optimal solution.
	Massive parameters of GA are essential to be controlled.	GA performs an excellent ability on parallel computing.
	GA is a time-consuming algorithm.	GA can increase the flexibility of searching for the optimal solution.
GSA	GSA is a time-consuming algorithm.	GSA is easy coding.
		It is affirmed that GSA can find the optimal solution.

**Table 5:** Tuned parameters of six types of ML models utilising GSA and GA.

Algorithms	Parameters	GA	GSA
ANN	Hidden layers	3	3
	Hidden neurons	20–10–5	20–10–5
	Learning rate	0.0663	0.1001
GBR	Depth_max_	86	90
	Split_min_	0.0001	0.01
	Learning rate	0.0947	0.4000
	Leaf_min_	57	21
DTR	Depth_max_	12	45
	Split_min_	9	16
	Leaf_min_	9	1
	Gain_min_	0.0775	0.3950
SVR	C_penalty_	25.9007	0.0001
	Epsilon	0.5621	0.0001
	Gamma	9.1228	10000.0000
RF	Depth_max_	86	64
	Split_min_	23	0.01
	Leaf_min_	57	17
	Gain_min_	56.4671	0.3950
kNN	k	4	11

**Table 6:** Fourteen types of GSA-GBR models with various numbers of inputs.

Inputs	GSA-GBRs
CM, FA, CT, W	GBR1
CM, FA, CT, W, WB	GBR2
CM, FA, CT, W, WB, S	GBR3
CM, FA, CT, W, WB, S, FAS	GBR4
CM, FA, CT, W, WB, S, FAS, SG	GBR5
CM, FA, CT, W, WB, S, FAS, SG, HM	GBR6
CM, FA, CT, W, WB, S, FAS, SG, HM, DOHM	GBR7
CM, FA, CT, W, WB, S, FAS, SG, HM, DOHM, FD	GBR8
CM, FA, CT, W, WB, S, FAS, SG, HM, DOHM, FD, FL	GBR9
CM, FA, CT, W, WB, S, FAS, SG, HM, DOHM, FD, FL, FTS	GBR10
CM, FA, CT, W, WB, S, FAS, SG, HM, DOHM, FD, FL, FTS, HC	GBR11
CM, FA, CT, W, WB, S, FAS, SG, HM, DOHM, FD, FL, FTS, HC, CW	GBR12
CM, FA, CT, W, WB, S, FAS, SG, HM, DOHM, FD, FL, FTS, HC, CW, CD	GBR13
CM, FA, CT, W, WB, S, FAS, SG, HM, DOHM, FD, FL, FTS, HC, CW, CD, TH	GBR14

## Data Availability

Data can be made available upon reasonable request.

## Abbreviations

**Abbreviations**	**Definition**
ASR	Aggregate Silicate Reaction
DTR	Decision Tree Regression
GBR	Gradient Boosting Regression
GSA	Grid Search Algorithm
GA	Genetic Algorithm
HP	Healing Performance
kNN	k-Nearest Neighbours
ML	Machine Learning
RF	Random Forest
R^2^	Coefficient of Determination
RMSE	Root Mean Square Error
SAP	Superabsorbent Polymers
SVR	Support Vector Regression
